# Promotion of women at the faculty of medicine at the University of Rostock: an archival case study

**DOI:** 10.1186/s12909-026-09969-z

**Published:** 2026-07-22

**Authors:** Sabrina Bindrich, Nico Wille

**Affiliations:** 1 Independent Researcher, Rostock, Germany; 2 University of Rostock, Department of Economics, Chair of Money and Credit, Rostock, Germany

**Keywords:** Gender equality, Women in medicine, Academic careers, Archival study

## Abstract

**Background:**

Women remain underrepresented in leadership positions in academic medicine, despite decades of formal equality policies. Historical institutional approaches can reveal persistent structural and cultural barriers. This study analyses archival material from the Medical Faculty of the University of Rostock to examine the development of institutional strategies supporting women in academic medicine.

**Methods:**

Archival sources, including faculty reports, correspondence, equality plans, and committee records from 1950 to 2002, were qualitatively analysed to reconstruct debates, measures, and perceived barriers. More recent material was gathered from institutional websites.

**Results:**

Early initiatives, including the 1962 Gender Equality Plan, combined support for women’s careers with scepticism about their scientific capabilities and family responsibilities. Subsequent plans introduced mentoring programmes, family-friendly policies, and gender-disaggregated monitoring. Despite these efforts, implementation varied across departments, and women remain underrepresented in senior positions.

**Conclusions:**

Institutional commitment to women’s advancement has increased over time, yet women remain underrepresented in senior academic positions. These findings suggest that formal equality measures alone may not be sufficient to achieve sustainable gender equality in academic medicine.

## Background

Several international studies and meta-analyses have demonstrated that women remain significantly underrepresented in leadership positions in academic medicine, particularly at the level of full professorships [1–4]. Despite increasing attention to gender equality in recent decades, structural barriers and persistent biases continue to shape academic career trajectories [5, 6].

Historically, women’s access to medical education and academic careers has been limited. In Prussia, women were only formally permitted to study at universities from 1908 onwards [7]. Early opposition to women’s participation in academic medicine was often grounded in medically framed arguments concerning biological differences, including menstruation, reproduction, and presumed intellectual inferiority [8]. Although such overt claims have largely disappeared, research suggests that women in academic medicine continue to face multiple structural challenges, including long qualification periods, exclusionary institutional practices, and persistent societal stereotypes regarding gender roles in healthcare professions [9].

While gender disparities in academic medicine are well documented, less is known about how institutional structures, attitudes, and promotion strategies have evolved over time within individual faculties. In particular, archival sources provide a unique opportunity to reconstruct historical developments, internal debates, and institutional responses that are not captured in contemporary quantitative analyses.

The University of Rostock, founded in 1419 and one of the oldest universities in Germany, provides a particularly valuable case for such an analysis. Archival records from the medical faculty document early discussions and measures related to gender equality, including a Gender Equality Plan from 1962 outlining women’s participation and proposed support structures [10–12]. Subsequent initiatives have continued to address structural challenges and promote women’s advancement, including mentoring programmes aimed at supporting female physicians in pursuing leadership positions [13–17]. While a complementary quantitative analysis of professorial careers at the University of Rostock is presented elsewhere [18], the present study focuses specifically on qualitative insights derived from archival sources. For contextual purposes, however, it is noteworthy that no women held full professorships prior to 1964. Although women constitute a majority in the lower academic tiers, they remain substantially underrepresented in senior academic positions. In 2018, women accounted for 63.16% of students, 59.69% of graduates from the faculty, 59.50% of newly qualified medical doctors, and 33.33% of habilitations, yet only 10.94% of professorships at the University of Rostock’s Faculty of Medicine were held by women. In comparison, the national average for female professors in medicine in Germany in 2018 was 23.69% [18]. Against this background, the present study analyses archival material from the Medical Faculty of the University of Rostock to examine the historical development of institutional approaches to the promotion of women in academic medicine. Particular attention is paid to internal debates, perceived barriers, and implemented measures, to better understand how gendered structures and strategies have evolved over time.

## Methods

This study is based on archival material from the University Archive of the University of Rostock. To identify relevant sources, the archive's online catalogue [19] was systematically searched using the keyword “Frauenförderung” (promotion of women). All records related to the Faculty of Medicine were screened for relevance. This search resulted in 11 archival files covering different periods and departments within the faculty.

The files were requested through the archive's access procedure and examined on site. All documents containing information on policies, measures, debates, or institutional strategies concerning the promotion of women were photographed and catalogued. In total, 40 documents were included in the analysis (Table 1). The corpus comprised faculty reports, internal correspondence, equality plans, committee records, and related administrative documents dating from 1950 to 1984. Material covering developments after 1984 was collected from publicly available documents on the University of Rostock website.

**Table 1:** Overview of archival documents related to women's promotion at the Faculty of Medicine, University of Rostock

Period	Number of documents
1960–1965	25
1972–1977	11
1979–1984	4
total	40

The documents were analysed qualitatively using a thematic approach. The analysis followed an inductive qualitative document analysis approach, combining systematic close reading with manual coding and thematic structuring. Following repeated close readings of the material, relevant passages were identified and summarized. Themes were developed inductively from the documents and subsequently organized according to historical periods and institutional contexts (e.g., faculty departments and committees). The analysis focused on reconstructing institutional strategies, policy developments, and debates concerning the promotion of women within the medical faculty. To ensure consistency, all documents were reviewed multiple times and interpretations were continuously compared across sources and periods.

To complement the qualitative analysis, archival records on student enrolment and graduation statistics were examined to trace the proportion of women at different academic levels within the Faculty of Medicine. Relevant files were identified through the archive catalogue using the keyword “Studentenzahlen” (student numbers). This search yielded 11 archival files, which were accessed and reviewed following the same procedure as the files on women's promotion. Quantitative data from eight relevant documents were extracted and compiled in a spreadsheet for descriptive analysis. The most recent archival source included was a report published in 2002, which provided a retrospective overview of developments since 1992. This analysis served as contextual background information and was not a primary focus of the study.

Unless otherwise indicated, all quotations from archival sources are the authors’ translations from the original German.

## Institutional context

The University of Rostock, founded in 1419 with papal approval, is one of the oldest universities in Germany. Its early history was marked by periods of instability, including temporary relocations and conflicts between the city and territorial rulers. A defining moment was the agreement of 1563 (*Formulae Concordiae*), which established a system of dual patronage between the ducal authorities and the city council, shaping the university’s governance and institutional structure for centuries [20].

Following phases of consolidation and reform, the university experienced periods of both prosperity and disruption, including the temporary division into two parallel institutions in Rostock and Bützow in the eighteenth century. In the nineteenth century, state consolidation and increased financial support led to renewed institutional stability and growth [21].

The twentieth century brought significant political transformations that also affected the university. Under National Socialism, academic self-governance was restricted [22], while the post-war period in the German Democratic Republic (GDR) was characterized by structural reforms and centralization. After German reunification in 1989, the university was reorganized according to the structures of the Federal Republic of Germany [23].

These historical developments provide the broader institutional context in which the medical faculty evolved and debates and measures concerning the role and promotion of women in academic medicine took place.

## Findings from the archive and institutional websites

The archival corpus consisted of two complementary datasets. The primary dataset comprised 40 documents related to women's promotion within the Faculty of Medicine (Table 1), including women's promotion plans, implementation reports, committee reports, administrative correspondence, and policy documents. These records covered the period from 1960 to 1984, with the largest concentration originating from the early 1960s and additional material from the 1970s and early 1980s.

A supplementary dataset consisted of eight documents containing enrolment, graduation, and institutional statistics used to trace the proportion of women at different academic levels within the University of Rostock up to 2002. These quantitative records covered the period from 1950 to 2002 and served as contextual information for interpreting long-term developments in women's participation in medical education and academic careers.

### Early documents and initial framing of women’s promotion (early 1960s)

In the early 1960s, archival documents primarily frame women’s participation in academic medicine in terms of workforce composition, structural support measures, and prevailing assumptions about gender roles.

The first official Gender Equality Plan mentioned in the archive files of the medical faculty dates to 1962. A faculty member from the medical school presented an accountability report on its implementation and outcomes. The report provides an overview of the involvement of women in the faculty: 2 of 11 lecturers, 7 of 35 non-habilitated senior consultants, and 96 of 227 junior doctors were female in 1962. In addition to outlining career prospects for female physicians, such as the planned habilitation of five senior female doctors, the plan proposed measures to ease women’s everyday responsibilities, for example, by establishing grocery shopping options, including an ordering system, at university medical facilities. Furthermore, existing childcare services, such as crèches, kindergartens, and after-school care, were expanded to better meet demand. These measures indicate an early institutional awareness of the structural barriers affecting women’s participation in academic medicine, particularly in relation to work–life balance [10].

In the same period, different specialties were asked to respond to a set of questions on the involvement of women in research. The questionnaire addressed respondents’ experiences with female physicians and asked, for example, *“what experiences have you had with women engaged in scientific work in your field?”* [11]. It further included normative and evaluative items such as whether *“women are capable of achieving the same scientific performance as men”*, whether it is *“good if women pursue scientific work”*, and whether it is *“possible for a wife”* or *“possible for a mother”* to engage in scientific work [11]. Additional questions asked respondents to assess *“for which scientific tasks women are particularly suitable or especially well suited”*, and conversely *“for which tasks women are not or are less suitable”* [11]. The questionnaire also included broader reflective items on whether *“real equality in all areas of life is possible”* and how the overall situation of early-career researchers was assessed within their departments [11]. One major concern was the perceived impact of pregnancy on workforce planning. A departmental statement noted that *“all married female physicians”* had been absent from work for extended periods due to pregnancy and complained that no physicians could be recruited for temporary replacement contracts [12]. Consequently, positions remained *“effectively vacant”* during maternity leave, and other staff members had to assume the additional work [12]. Furthermore, scientific work was described as taking place largely outside regular working hours, which conflicted with expectations that women fulfil responsibilities as mothers and caregivers. In addition, doubts were expressed as to whether women could achieve the same level of scientific performance as men, with one department explicitly stating that *“truly creative talent and achievement by a woman should be regarded as a rarity”* and arguing that women’s strengths *“naturally lie in organizing family life and raising children”* [12]. Nevertheless, it was acknowledged that women with particular aptitude for scientific work should be supported [12]. This suggests a conditional acceptance of women in academia, in which support was granted selectively rather than as a general principle. The Department of Obstetrics and Gynaecology went further by explicitly questioning the rationale of promoting women in academic careers. One report stated that *“the question arises whether the special promotion of women is meaningful for the interests of university clinics”* and argued that *“the scientifically active man will ultimately be of greater benefit to us than, in general, the scientifically active woman”* [11]. At the same time, even this department endorsed social measures to facilitate women’s participation [11], highlighting the coexistence of support and scepticism within the same institutional context.

Overall, early documents combine emerging institutional support measures with strong assumptions about gendered role divisions, particularly regarding women’s compatibility with scientific careers.

### Departmental implementation and ideological framing (1963–early 1970s)

From 1963 onwards, women’s promotion was implemented through the development of discipline-specific plans at the departmental level, while simultaneously being framed within the broader ideological and legal discourse of the GDR.

For the years 1963 and 1964, individual academic departments were required to develop their own discipline-specific plans for the promotion of women [13]. In the GDR, gender equality was framed as a central societal principle, as reflected in the SED communiqué *Women, Peace, and Socialism*, which is frequently referenced in these documents [24, 25]. Archival records explicitly linked women's promotion to state policy, stating that public authorities and institutional leaders were obliged to “create all conditions enabling women to participate in the work process and to develop their creative abilities” [24]. These references suggest that faculty-level women's promotion plans were closely aligned with the ideological and legal framework of the GDR. Accordingly, the plans emphasised further education, professional qualification, and academic advancement, alongside measures to reduce everyday burdens [13, 24–31]. These recurring elements suggest a strong alignment between institutional policies and broader state ideology. At the same time, the extent and focus of these measures varied between departments. While some units proposed structured career development strategies, others, such as the department of obstetrics and gynaecology, did not include specific measures for women in academia and instead focused on mid-level personnel [32]. This variation indicates that the implementation of equality policies remained uneven and was influenced by local disciplinary cultures. It is also noteworthy that preventive cancer screening was included in some plans, reflecting a broader understanding of women’s health and wellbeing in the workplace context [29, 33].

Compared to the initial faculty-wide framework of 1962, this period is characterized by the decentralization of implementation to individual departments and a strong embedding of women’s promotion within state socialist equality ideology.

### Consolidation and intensification of monitoring (1970s–1984)

Building on earlier reporting structures introduced in the 1960s, the following decades show a consolidation and intensification of monitoring and evaluation practices in women’s promotion.

The archival records further include progress reports from individual departments on the implementation of their women’s advancement plans [34–44], indicating that these measures were subject to regular monitoring. However, these reports also highlight persistent challenges. A recurring issue was the lack of sufficient day care facilities for small children [36, 38, 43], which directly affected women’s ability to participate fully in academic work. In addition, the Faculty of Medicine reported that theoretical medical institutes appeared to pay little attention to the women’s advancement plans [45], pointing to inconsistencies in institutional engagement. A resolution of the party leadership in the field of medicine from October 1984 called for intensified and systematic monitoring of women's advancement. The document stated that *“regular control of women's promotion within the field of medicine”* should be ensured and that clinics and institutes should devote *“increased attention”* to the development of female scientists [46]. Particular emphasis was placed on academic qualification pathways. The resolution demanded intensified support for women pursuing *Promotion B* degrees (in the GDR roughly equivalent to the current German habilitation) and called for targeted discussions with female early-career academics to establish and monitor *“concrete support measures and timelines”* [46]. In addition, the Women's Commission was instructed to organize discussion forums with female doctoral candidates to address *“the status and problems of their scientific work”* and to promote the exchange of experiences [46]. These measures suggest that gaps between policy and practice were recognized at higher institutional levels and that institutional attention had shifted from general questions of women's participation toward the promotion of female academic careers and leadership development. In contrast to earlier decades, where monitoring practices were still embedded within broader planning documents, this period reflects a more systematic and institutionally coordinated form of oversight.

### Contemporary institutionalization of gender equality (2004–2021)

In the contemporary period, gender equality policies at the University of Rostock are characterized by formalized institutional frameworks and structured career development instruments.

In this period, the University of Rostock continues to implement comprehensive strategies for the promotion of women and gender equality. The adoption of a university-wide gender equality policy in 2004 marked a significant step toward formalizing these efforts [14]. Key measures include gender-sensitive job advertisements, preferential consideration of women in cases of equal qualification, and targeted support for underrepresented areas. Structural measures such as part-time career options, family-friendly working conditions, and the expansion of childcare support reflect a continued focus on reconciling academic careers with family responsibilities. These developments indicate a shift toward more systematic and institutionalized approaches to gender equality.

Subsequent women’s advancement plans further expanded these measures by introducing gender-disaggregated data collection, the cascade model for increasing female representation, and structural changes to recruitment and career development processes [15–17]. At the same time, mentoring programmes and targeted support for female students and early-career researchers have become increasingly prominent [17]. Despite these efforts, recent reports continue to highlight challenges, particularly the loss of women along the academic career pipeline [47] and the underrepresentation of women in senior academic positions [17]. This suggests that, while institutional commitment to gender equality has increased over time, structural barriers to women’s advancement persist.

Since May 2021, a mentoring programme for female physicians aiming for leadership positions has been implemented by the University of Rostock and the medical faculty. The programme supports participants in planning their academic careers through one-on-one mentoring [48].

Compared to earlier periods, contemporary policies reflect a high degree of institutionalization, with a stronger focus on structural equality mechanisms and individual career support, although persistent disparities remain.

**Fig. 1:**
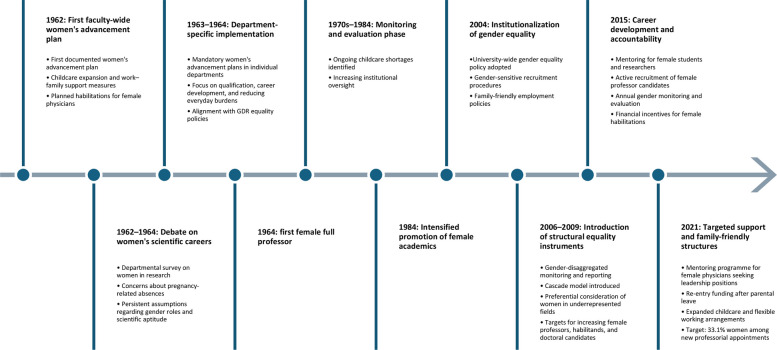
Timeline of major women's advancement policies and institutional developments at the Faculty of Medicine, University of Rostock

To provide a clear overview of the developments discussed above, reference is made to Fig. 1.

## Discussion

The present study provides archival insights into the historical development of institutional approaches to the promotion of women at the Medical Faculty of the University of Rostock. Overall, the findings reveal a persistent gap between increasing institutional support for women’s advancement and the continued underrepresentation of women in senior academic positions. While archival sources from earlier decades documented explicit scepticism regarding women’s roles in academic medicine, more recent documents suggest that structural factors may contribute to the persistence of gender inequalities despite formal equality measures.

A central finding of this study is the ambivalence of early institutional attitudes. While the 1962 Gender Equality Plan and subsequent measures clearly aimed to support women’s participation in academic medicine, the archival material simultaneously documents substantial doubts about women’s scientific capabilities and their ability to reconcile professional and family responsibilities. This coexistence of support and scepticism suggests that early promotion efforts were not grounded in a fully accepted principle of gender equality, but rather in a conditional framework in which women’s participation was supported only under certain circumstances. Such ambivalence reflects broader societal norms of the time and highlights the influence of traditional gender roles on institutional decision-making.

The findings from the period of the German Democratic Republic further illustrate the complex relationship between formal policy and institutional practice. While gender equality was explicitly promoted as a political and social goal, and comprehensive measures for the advancement of women were implemented, the archival records reveal considerable variation in how these policies were interpreted and applied across departments. This suggests that top-down equality frameworks did not automatically translate into uniform institutional practices. Instead, local disciplinary cultures and individual attitudes continued to shape the extent to which women were actively supported in their academic careers. The introduction of targeted initiatives like mentoring programs reflects a growing emphasis on individualized support mechanisms, complementing broader structural measures in the contemporary period.

Across the different time periods examined, a notable continuity can be observed in the structural challenges affecting women’s careers. Issues such as the compatibility of academic work with family responsibilities, particularly in relation to childcare, appear consistently in the archival material from the 1960s through to the present. Although institutional responses have become more comprehensive and formalized over time, including the expansion of childcare services, flexible working arrangements, and mentoring programmes, these measures have not fully eliminated barriers to women’s advancement. The persistence of such challenges is also reflected in more recent data indicating a continued loss of women along the academic career pipeline and their underrepresentation in senior positions [17].

These findings are consistent with existing research on gender disparities in academic medicine, which highlights structural barriers, long qualification periods, and implicit biases as key factors influencing women’s career progression [1–4, 9]. At the same time, the present study extends this literature by providing a longitudinal, institution-specific analysis of equality policies, support measures, and documented debates surrounding women’s academic careers. In contrast to cross-sectional or quantitative studies, the use of archival material allows for a reconstruction of internal debates and decision-making processes, thereby offering a more nuanced understanding of institutional dynamics.

The study has several strengths. The use of extensive archival sources enables a detailed reconstruction of institutional practices and attitudes over an extended historical period. This long-term perspective provides insights into both continuity and change in approaches to gender equality that would not be accessible through contemporary data alone.

At the same time, certain limitations must be acknowledged. The analysis focuses on a single institution and is therefore not directly generalizable to other contexts. In addition, archival sources reflect institutional perspectives and may not fully capture the experiences of individual women affected by these policies. Furthermore, the selection and availability of documents may influence the findings. The study also does not provide a longitudinal quantitative analysis of women’s representation in academic leadership positions, nor does it systematically measure changes in attitudes, implicit bias, or other cultural factors over time. Consequently, conclusions regarding the persistence of gender inequalities must be interpreted primarily in relation to documented institutional policies and structures rather than as direct evidence of individual experiences or attitudes.

Despite these limitations, the study has important implications. It highlights the need to consider not only formal equality policies, but also the underlying institutional cultures and assumptions that shape their implementation. The findings suggest that sustainable progress in promoting women in academic medicine requires not only structural measures but also ongoing reflection on the historical and cultural factors that have shaped gender inequalities within academic institutions. Future research could build on these findings by combining archival approaches with quantitative analyses and qualitative interviews to further explore the relationship between institutional policies and individual career trajectories.

## Conclusion

The archival analysis of the Medical Faculty of the University of Rostock highlights the long-term evolution of institutional approaches to promoting women in academic medicine. While early efforts were marked by ambivalence and conditional support, formal policies and measures have become increasingly comprehensive over time, including mentoring programmes, flexible career paths, and family-friendly initiatives. Despite these advances, women remain underrepresented in senior academic positions. These findings suggest that formal equality measures alone may not be sufficient to achieve sustainable gender equality in academic medicine. Such insights can inform more effective and context-sensitive strategies for achieving gender equality in academic medicine.

## Data Availability

The data that support the findings of this study are available from the University of Rostock. Access to the archival materials can be requested via the University of Rostock archive catalogue and access system [19]. Upon approval, the documents can be consulted on site at the University Archive of the University of Rostock. Data are however available from the authors upon reasonable request and with permission of the University of Rostock. A complete list of all archival files analysed in this study is provided in the appendix.

## Appendix

**Table 2:** Appendix Table 1: Search results from the University of Rostock Archives - Search query: women's advancement / promotion of women (“Frauenförderung”)

**Archive title (German)**	**English ** **translation**	**Department / Unit**	**Archive reference**	**Date range**
Frauenförderungsplan	Women's Promotion Plan	Department of Obstetrics and Gynaecology (Frauenklinik)	0813	1963–1964
Frauenförderung	Promotion of Women	Dean's Office (Dekanat) / Faculty Administration (Bereichsleitung)	1097	1962–1973
Frauen- und Jugendförderung	Promotion of Women and Youth	Dean's Office (Dekanat) / Faculty Administration (Bereichsleitung)	1160	1974–1977
Frauenförderung	Promotion of Women	Dean's Office (Dekanat) / Faculty Administration (Bereichsleitung)	1179	1979–1984
Frauenförderung	Promotion of Women	Department of Medicine (Medizinische Klinik)	1335	1960–1964
Jugend- und Frauenförderung	Promotion of Youth and Women	Department of Paediatrics (Kinderklinik)	1534	1974–1978
Revision, Planung, Arbeitsberatungen, Frauenförderung	Audits, Planning, Staff Meetings, and Promotion of Women	Department of Dermatology (Hautklinik)	1549	1949–1975
Frauenförderung	Promotion of Women	Department of Neurology and Psychiatry (Nervenklinik)	1659	1955–1964
Jugend- und Frauenförderung	Promotion of Youth and Women	Department of Orthopaedics (Orthopädische Klinik)	1744	1963–1975
Frauenförderung	Promotion of Women	Dental Clinic (Zahnklinik)	1791	1963–1975
Jugendförderung an der Medizinischen Fakultät	Youth Promotion at the Faculty of Medicine	Dean's Office (Dekanat) / Faculty Administration (Bereichsleitung)	1839	1959–1964

**Table 3:** Appendix Table 2: Search results from the University of Rostock Archives - Search query: number of students (“Studentenzahlen”)

**Archive title (German)**	**English ** **translation**	**Department / Unit**	**Archive reference**	**Date range**
Erziehung und Ausbildung	Education and Training	Rectorate (Rektorat ab 1945)	0184	1988–1989
„Eckzahlen der Universität Rostock“	Key Figures of the University of Rostock	Rectorate (Rektorat ab 1945)	0516	1985
Perspektivplanung der Universität	Strategic Development Planning of the University	Rectorate (Rektorat ab 1945)	0901	1959–1967
Direktiven zum Volkswirtschaftsplan	Directives for the National Economic Plan	Rectorate (Rektorat ab 1945)	0902	(1950), 1960–1962
Erfüllungsanalysen und Direktiven zum Volkswirtschaftsplan der Universität	Performance Analyses and Directives for the National Economic Plan of the University	Rectorate (Rektorat ab 1945)	0904	1974–1981
Ministerium für Hoch- und Fachschulwesen: Vergleich ausgewählter Zahlen und Fakten der Universitäten und Hochschulen	Ministry of Higher and Technical Education: Comparison of Selected Figures and Facts of Universities and Higher Education Institutions	Rectorate (Rektorat ab 1945)	0937	1981–1985
Perspektivplanvorschlag (1960–65) zu Studienangelegenheiten	Strategic Planning Proposal (1960–65) for Academic Affairs	Rectorate (Rektorat ab 1945)	1008	1959
"Rückblick und Verbleib – Rostocker Absolventinnen und Absolventen 1996–1999" (Druck)	Retrospective and Career Paths of Rostock Graduates 1996–1999 (Print)	n/a	3399	2002
Übersichten Studentenzahlen und belegte Fächer	Overviews of Student Numbers and Subjects Enrolled	Faculty of Humanities (Philosophische Fakultät)	024	1946–1968
Stellenplanung/Kaderentwicklung	Staff Planning and Personnel Development	Institute of English Studies (Institut für Anglistik)	353	1959–1970
Planung und Statistik: Studentenzahlen	Planning and Statistics: Student Numbers	n/a	156	1950–1968
